# Evaluation of abdominal compression band for pediatric postural tachycardia syndrome: a crossover study

**DOI:** 10.3389/fped.2025.1573091

**Published:** 2025-05-22

**Authors:** Ginroku Yamawake, Seiji Yoshida, Hidetaka Tanaka, Yusuke Kurooka, Atsuko Kubo, Yoshitaka Ota, Midori Mizutani, Akira Ashida

**Affiliations:** ^1^ Department of Pediatrics, Osaka Medical and Pharmaceutical University Hospital, Takatsuki, Japan; ^2^ Tanaka OD Hypotension Clinic, Osaka, Japan; ^3^ Department of Pediatrics, Saiseikai Ibaraki Hospital, Ibaraki, Japan; ^4^ Department of Pediatrics, Saiseikai Suita Hospital, Suita, Japan; ^5^ Department of Pediatrics, Hokusetsu General Hospital, Suita, Japan

**Keywords:** postural tachycardia syndrome, abdominal compression band, children, cardiovascular autonomic function, hemodynamic

## Abstract

**Background:**

Abdominal compression is an effective nonpharmacological treatment for orthostatic intolerance. Although the efficacy of abdominal compression bands has been studied in adults, studies involving children are limited. This study investigates the efficacy of abdominal compression bands in children diagnosed with postural tachycardia syndrome (POTS), using a larger cohort and incorporating autonomic nervous function assessment via frequency analysis.

**Methods:**

A crossover study was conducted with 23 patients with POTS (mean age 13.4 ± 0.8 years), with and without abdominal compression bands (20 mmHg). Standing symptoms, hemodynamics (blood pressure, heart rate [HR], cardiac index [CI], stroke volume [SV], and total peripheral resistance [TPR]), and cardiovascular autonomic function were assessed by spectrum analysis during standing tests. These variables were compared with and without bands.

**Results:**

When standing with the abdominal compression band, subjective symptoms improved, increases in heart rate (HR) and decreases in stroke volume (SV) were suppressed, blood pressure was maintained, and cardiac parasympathetic function was improved. By contrast, in the supine position, patients showed an increased HR, decreased CI, increased TPR, lower cardiac parasympathetic function, and higher cardiac sympathetic function.

**Conclusion:**

Abdominal compression bands may relieve symptoms while standing and improve hemodynamic and autonomic nervous functions in children with POTS.

## 1 Introduction

Orthostatic intolerance refers to a spectrum of disorders involving autonomic dysfunction, including postural tachycardia syndrome (POTS) and orthostatic hypotension (OH), which are associated with severe autonomic failure (1).

In Japan, orthostatic dysregulation (OD) is often used synonymously with orthostatic intolerance. Approximately 10% of junior high and high school students are estimated to suffer from OD, a leading cause of school absenteeism (2).

The pathophysiology of OD arises from impaired compensatory regulatory mechanisms that manage the dynamic circulatory changes during orthostasis. These mechanisms include circulating blood volume, cardiac output, peripheral vascular characteristics, cerebral circulatory regulatory characteristics, and autonomic nervous system integration. OD is a functional physical disorder caused primarily by autonomic nervous system dysfunction, leading to abnormal regulation of circulation (3). The diagnosis and treatment of OD in Japanese children are guided by the recommendations of the Japanese Society of Psychosomatic Pediatrics. These guidelines prioritize guidance and education for parents and children, non-pharmacological treatments, contact with school personnel, medication, strategies of psychosocial intervention, and psychotherapy, with nonpharmacological therapy being as important as disease education (4).

Many OD practice guidelines recommend therapeutic orthotics, such as abdominal compression bands and compression socks, as nonpharmacological therapies (5, 6). Studies have reported an improved orthostatic tolerance to abdominal compression in adult patients (7, 8). Patients with POTS, a subtype of OD, have been shown to have increased venous pooling in the lower extremities and intra-abdominal organs during orthostasis, particularly in the intra-abdominal organs rather than in the lower extremities (8, 9). Therefore, abdominal compression bands, which aim to increase venous return by externally applying pressure to blood pooling in the intra-abdominal organs, are considered effective. However, their effectiveness in children has only been reported in nine cases conducted by Tanaka (10).

In this study, we aimed to perform a crossover analysis with and without abdominal compression bands, involving a larger pediatric population compared to previous studies. Additionally, we assessed autonomic nervous system function using frequency analysis to further examine the efficacy of abdominal compression bands in children with POTS.

## 2 Materials and methods

### 2.1 Participants

Twenty-three patients (13 boys and 10 girls, mean age: 13.4 ± 0.8 years, mean BMI-SD: −0.9 ± 0.2) aged 12–15 years were included in this study. They visited the Department of Pediatrics at Osaka Medical and Pharmaceutical University Hospital between October 2023 and August 2024 and were diagnosed with POTS based on the diagnostic criteria of the Japanese Society of Psychosomatic Pediatrics at the initial standing test before starting pharmacotherapy. Common underlying diseases, including iron deficiency anemia and thyroid dysfunction, were excluded from the blood tests.

### 2.2 Study design

**Figure 1:**
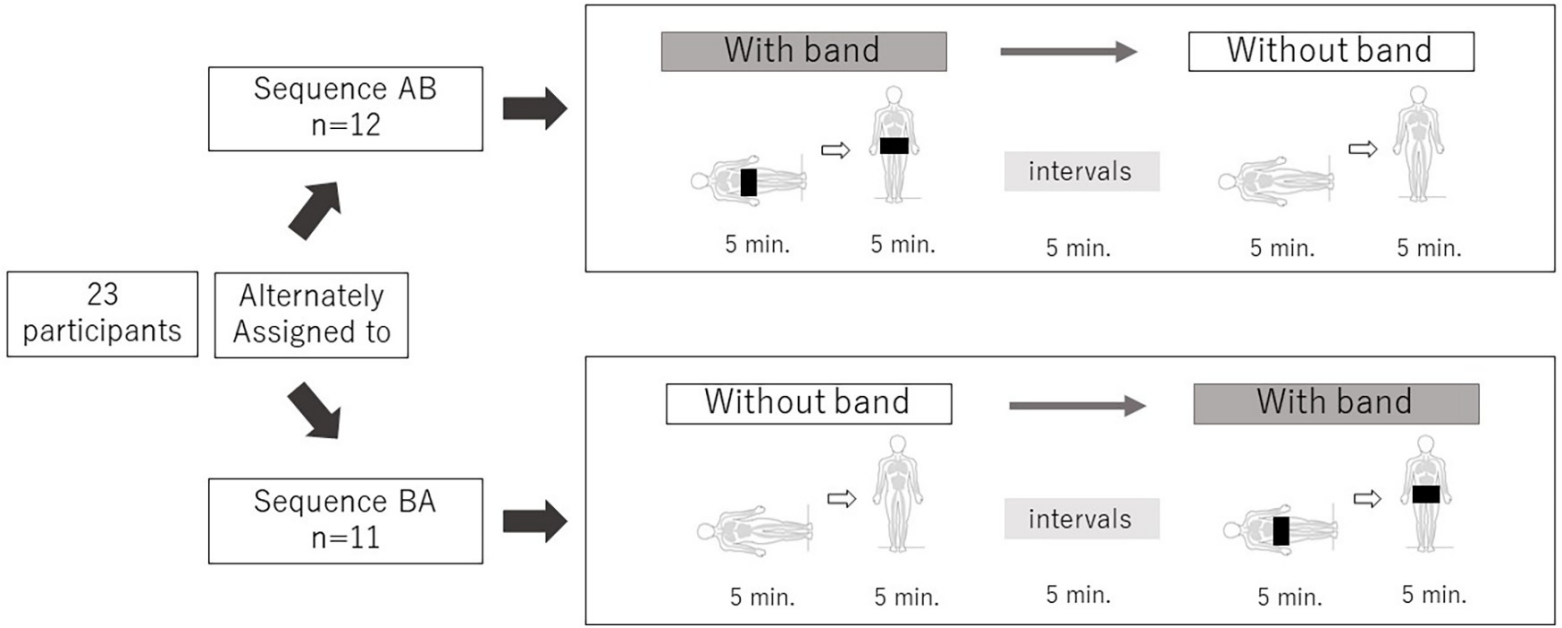
A crossover study; 5 min each in the supine and standing positions. 23 participants were alternately assigned to sequence AB/BA. Each standing test was performed at a 5 min interval, with and without the abdominal compression band.

**Figure 2:**
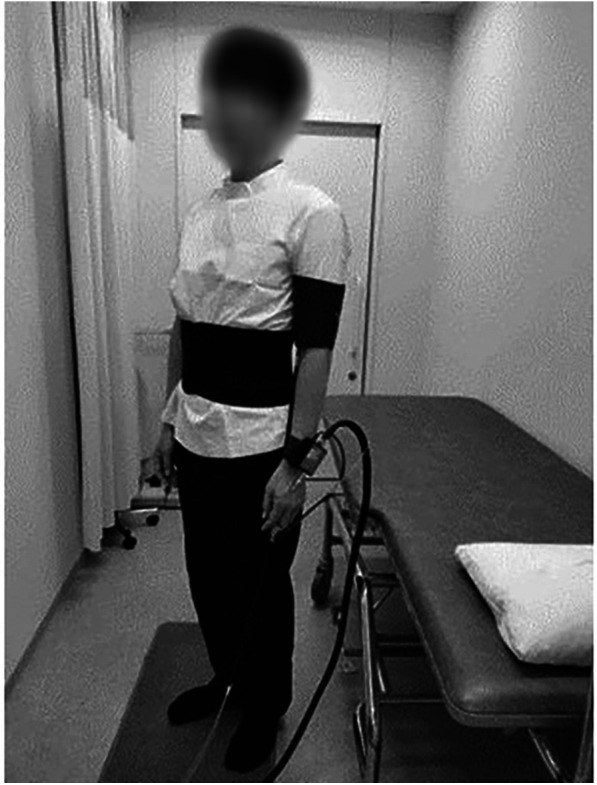
A standing test with the abdominal compression band.

A two-group alternating crossover AB/BA design (Figure 1) was used to evaluate the effect of the abdominal compression band. Each patient underwent two consecutive standing tests in the morning. Participants were alternately assigned to an AB or BA sequence. The group assigned to AB underwent a standing test in the supine and standing positions for 5 min each with the abdominal compression band (Figure 2), followed by a 5 min interval. After the first standing test and an interval, the second standing test was performed in the supine and standing positions for 5 min each without the band. Similarly, the participants assigned to BA performed the first standing test without the band, took an interval and performed the second standing test with the band.

**Figure 3:**
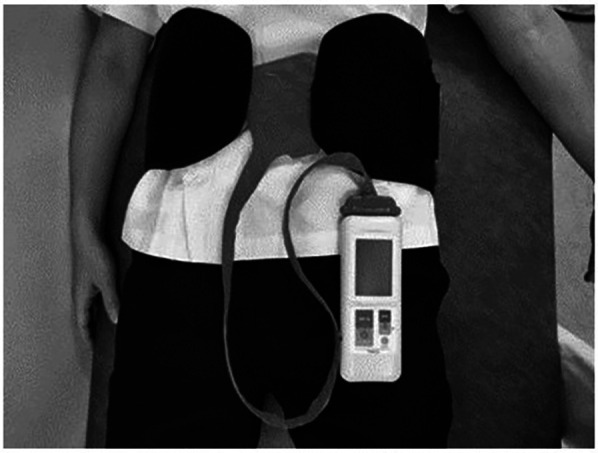
The abdominal compression band and a portable contact pressure measuring device.

The abdominal compression band (made by ATERCEL, China) measured 19.5 cm in width. A portable contact pressure measuring device (PalmQ, CAPE, Kanagawa, Japan) was placed under the abdominal band at a pressure to 20 mmHg (Figure 3).

The evaluation variables were standing-related symptoms, hemodynamics (blood pressure (BP), heart rate (HR), cardiac index (CI), stroke volume (SV), and total peripheral resistance (TPR)), and cardiovascular autonomic function in the standing test and were compared with and without wearing the abdominal compression band.

Each evaluation parameter is described as follows:

#### 2.2.1 Standing symptoms

Five symptoms—dizziness, blurred vision, headache, palpitations, and fatigue—were evaluated through participant interviews conducted after the test. Symptom intensity was rated on an 11-point visual analog scale ranging from 0 (no symptoms) to 10 (severe symptoms).

#### 2.2.2 Hemodynamics

**Figure 4:**
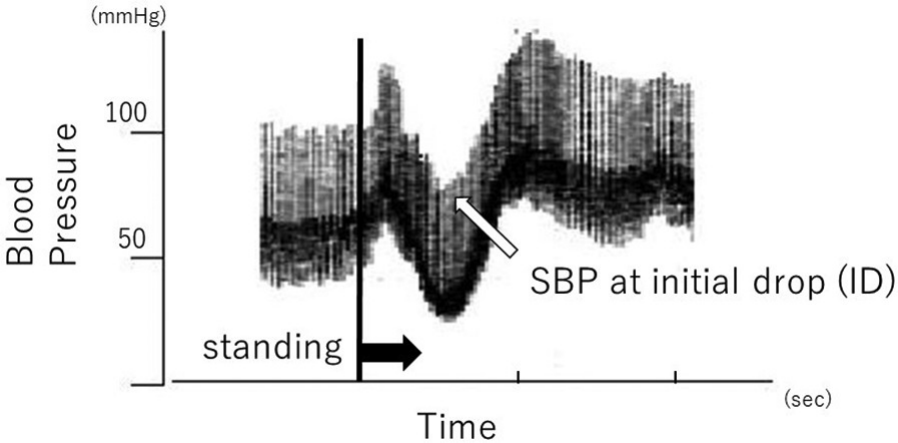
Change in blood pressure after standing.

Systolic blood pressure (SBP) in the supine position, at initial drop (ID) and during standing were measured using continuous noninvasive finger arterial pressure measurement (Finapres, FMS, Amsterdam, The Netherlands). ID is defined as the initial drop in SBP immediately after standing (Figure 4). Muscle contractions of the lower extremities and abdomen while standing cause a temporary increase in venous return and sudden increases in right atrial pressure. The baroreceptor reflex then decreases systemic vascular resistance, resulting in ID. ID in SBP occurs in the first 5–15 s on standing, and a transient fall of up to 40 mmHg SBP is considered normal (11). ID is related to dizziness and blurred vision.

HR, CI, SV, and TPR were also measured as well as SBP. The SBP, HR, CI, SV, and TPR were averaged from 3–5 min after standing.

The amount of change (standing value minus supine value) was calculated for SBP and HR, which were defined as ΔSBP and ΔHR each. The rate of change (standing value divided by supine value) was calculated for CI, SV, and TPR.

In addition, since it is possible that body size may affect the change in venous return with the abdominal compression band, we evaluated the correlation between the BMI-SD and the rate of change in SV, which is one of the indices reflecting venous return. The rate of change in SV was calculated dividing the change in SV with the band by the change in SV without the band.

#### 2.2.3 Autonomic nervous function

Heart rate and blood pressure variability during the standing test were evaluated by frequency analysis. Two frequency components were evaluated: the high-frequency (HF; 0.15–0.4 Hz) and low-frequency (LF; 0.04–0.15 Hz) components.

It is generally accepted that the HF component of RR interval variability (RR-HF) is mediated by cardiac parasympathetic tone generated by respiration, whereas the LF component of RR interval variability (RR-LF) is mediated by both cardiac sympathetic and parasympathetic tone. The ratio of the LF to HF components of RR interval variability (RR-LF/HF) is considered an index of cardiac sympathetic tone (12, 13). In contrast, the LF component of BP variability has been reported to parallel sympathetic vasomotor activity (12, 14). Therefore, we used the LF component of DBP variability (DBP-LF) to evaluate sympathetic vasomotor activity.

A Finometer cuff (model 1; FMS, Amsterdam, The Netherlands) was placed on the middle phalanx of the third finger on the right hand. To insure optimal Finometer BP measurements, we used appropriate cuff sizes (S or M) according to the manufacturer's instructions. Spectral analysis was conducted using the maximum entropy method (MemCalc for Windows version 1.2; Suwa Trust, Tokyo, Japan) on the time-series data from 3–5 min for each variable.

### 2.3 Statistical analysis

Corresponding paired tests were conducted on measurements before and after the intervention. The Wilcoxon signed-rank test was used owing to the lack of normality in the aggregated data. Statistical significance was set at *p* < 0.05. Data were summarized using medians and interquartile ranges. All statistical analyses were conducted using JMP version 17 (SAS Institute, Cary, NC, USA).

## 3 Result

**Figure 5:**
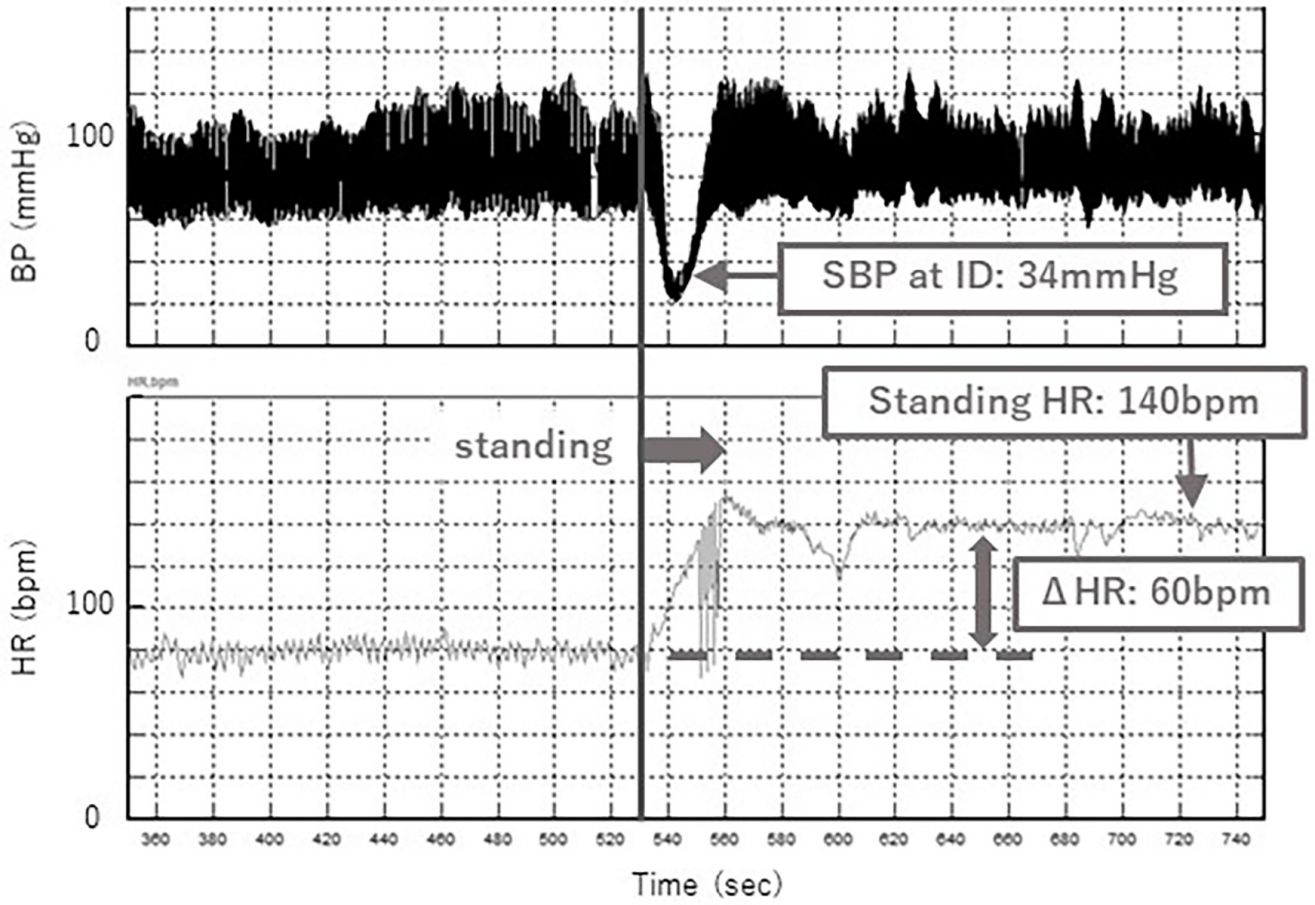
Change in SBP at ID and HR while standing without band.

**Figure 6:**
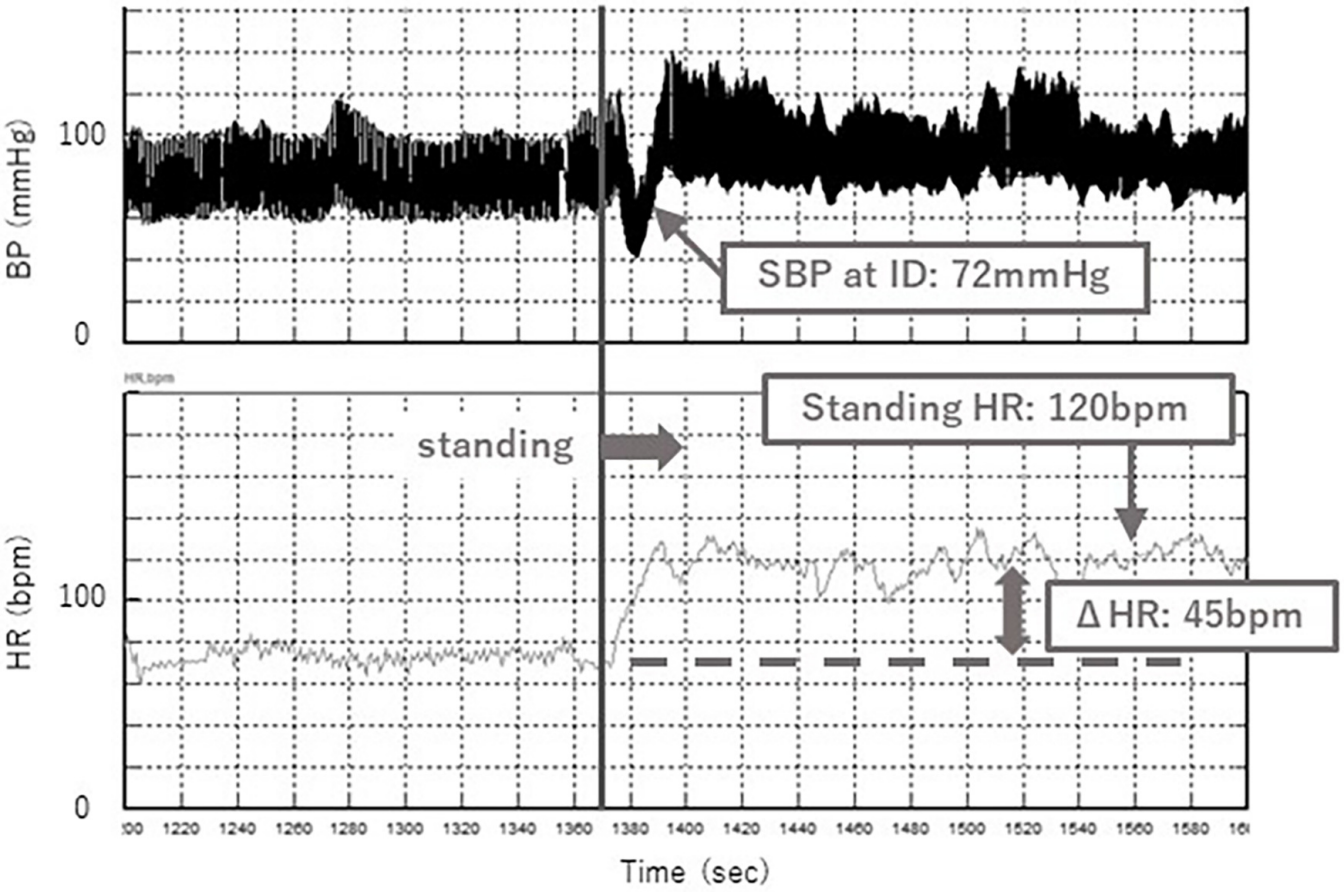
Change in SBP at ID and HR while standing with band.

A typical case of the standing test with and without the abdominal compression band is shown in Figures 5, 6. Without the band, a decrease in SBP at ID and an increase in HR were observed after standing up (Figure 5), while these changes were significantly suppressed with the band (Figure 6). Orthostatic symptoms were also markedly improved, and the visual analog scale of headache in this patient improved from 7/10 without the band to 1/10 with the band.

### 3.1 Standing symptoms

When wearing the abdominal compression band, standing-related symptoms (dizziness, blurred vision, headache, palpitations, and fatigue) improved significantly compared with when not wearing it (Table 1).

**Table 1:** Comparison of standing symptoms.

	With band	Without band	*p*
Dizziness	2 (1–5)	5 (2–8)	<0.01
Blurred Vision	0 (0–2)	4 (0–6)	<0.01
Headache	1 (0–3)	3 (0–7)	<0.01
Palpitations	1 (0–5)	5 (0–7)	<0.01
Fatigue	4 (1–6)	6 (2–7)	<0.05

Each orthostatic symptom was rated on a visual analog scale. Values represent the median of 23 subjects, and quartile ranges are shown in parentheses.

### 3.2 Hemodynamics

SBP in the supine position was not significantly different with and without the band. Change in SBP (ΔSBP) was significantly greater with the band (Table 2).

**Table 2:** Comparison of hemodynamics.

	With band	Without band	*p*
su SBP (mmHg)	102 (94–111)	105 (97–110)	0.20
ΔSBP	14 (3–18)	5 (−5–10)	<0.05
SBP at ID (mmHg)	76 (69–84)	67 (60–80)	<0.01
su HR (bpm)	83 (74–90)	80 (73–90)	<0.05
st HR (bpm)	118 (105–124)	120 (116–131)	<0.01
ΔHR	30 (25–36)	41 (34–48)	<0.01
su CI (L/min/m²)	2.2 (2.0–2.5)	2.5 (2.2–2.8)	<0.01
st CI (L/min/m²)	2.3 (2.1–2.5)	2.4 (2.1–2.6)	0.32
Change in CI	1.05 (0.92–1.12)	0.96 (0.87–1.10)	<0.05
su SV (ml)	39.6 (33.0–45.6)	47.4 (35.8–51.5)	<0.01
st SV (ml)	30.8 (24.9–33.5)	29.3 (22.8–32.9)	<0.05
Change in SV	0.76 (0.68–0.85)	0.64 (0.60–0.70)	<0.01
su TPR (mmHg.sec/ml)	1.5 (1.3–1.6)	1.4 (1.1–1.5)	<0.01
Change in TPR	1.08 (1.06–1.15)	1.13 (1.00–1.23)	0.29

Values represent the median of 23 subjects; figures in parentheses indicate the interquartile range. su, supine; st standing; SBP, systolic blood pressure; ID, initial drop; HR, heart rate; CI, cardiac index; SV, stroke volume; TPR, total peripheral resistance.

ID was significantly higher with the band.

**Figure 7:**
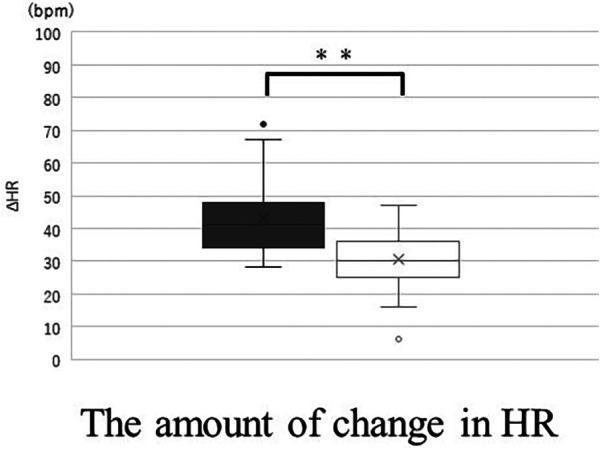
The amount of change (standing value minus supine value) in HR without band (▪) and with band (□). Box plot, median (IQR), mean values (×), with outliers. **p < 0.01. HR, heart rate.

**Figure 8:**
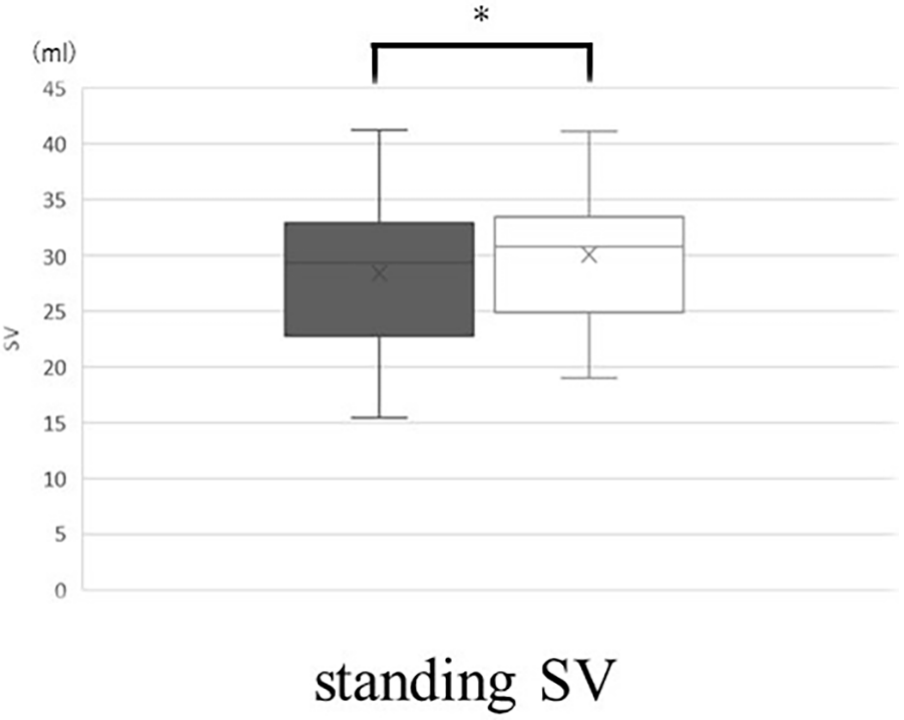
The values in standing SV without band (▪) and with band (□). Box plot, median (IQR), mean values (×). *p < 0.05. SV, stroke volume.

Supine HR was significantly higher with the band and HR while standing was significantly higher without the band. The change in HR (ΔHR) was significantly smaller with the band (Figure 7). The CI in the supine position was significantly smaller with the band, but the CI in standing position is no significantly different with and without the band. For SV, the values with the band were significantly lower than those without the band in the supine position, however, the values with the band were significantly greater than those without the band in the standing position (Figure 8). There was no correlation between BMI-SD and the rate of change in stroke volume (*p* *=* 0.91). Supine TPR was significantly higher with the band, and the rate of change in TPR was not significantly different between the two groups.

### 3.3 Frequency analysis

**Figure 9:**
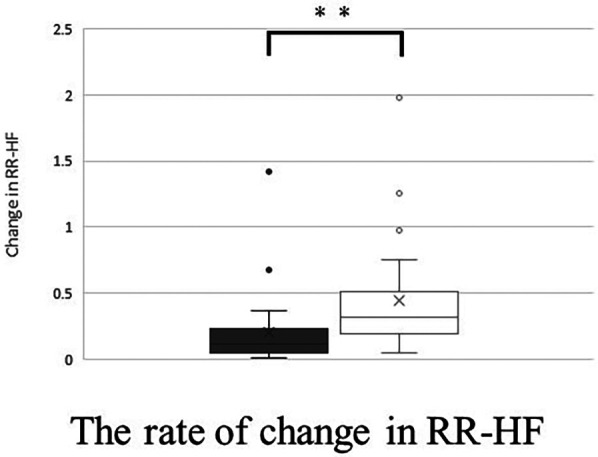
The rate of change (standing value divided by supine value) in RR-HF without band (▪) and with band (□). Box plot, median (IQR), mean values (×), with outliers. **p < 0.01.

For RR-HF, RR-HF in the supine position showed a significant decrease with the band. RR-HF change in the standing position was significantly higher with the band (Figure 9) (Table 3).

**Table 3:** Comparison of autonomic nervous function.

	With band	Without band	*p*
su RR-HF (msec^2^)	176.3 (118.5–493.2)	318.4 (139.7–771.0)	<0.01
Change in RR-HF	0.32 (0.20–0.51)	0.11 (0.05–0.23)	<0.01
su RR-L/H	1.48 (0.56–3.79)	1.24 (0.49–1.67)	<0.05
Change in RR-LF/HF	1.85 (1.03–4.74)	2.96 (1.17–6.33)	0.10
su DBP-LF (mmHg^2^)	3.37 (2.28–5.90)	3.00 (1.66–4.89)	0.93
Change in DBP-LF	2.24 (1.52–4.60)	2.77 (1.80–3.66)	0.64

Values represent the median of 23 subjects; figures in parentheses indicate the interquartile range. su, supine; RR-HF, high-frequency component of RR interval variability; RR-LF/HF, ratio of the LF to HF components of RR interval variability; DBP, diastolic blood pressure; DBP-LF, low-frequency component of DBP variability.

For RR-LF/HF, RR-LF/HF in the supine position was significantly higher with the band. However, there was no significant difference between the two groups in terms of RR-LF/HF changes during standing.

Regarding LF-DBP, there were no significant differences in the rate of change of LF-DBP in the supine position and LF-DBP in the standing position with or without the band.

## 4 Discussion

This study evaluated the efficacy of the abdominal compression band in pediatric patients with POTS and examined its effects on standing, hemodynamics, and autonomic nervous function.

When a person stands, approximately 500–800 ml of blood moves to the abdomen and lower extremities. As a normal response, the autonomic nervous system (sympathetic and parasympathetic) is activated to maintain blood pressure through increased HR and peripheral vasoconstriction. Simultaneously, the abdominal and lower extremity muscles rhythmically contract to compress the capillary vessels and maintain venous return (15). By contrast, the pathophysiology of POTS involves the inability to maintain adequate venous return in an standing position, leading to compensatory tachycardia (16). The pathogenesis of POTS varies widely and is classified into the following subtypes: hyperadrenergic, neuropathic, hypovolemic, and autoimmune POTS. These mechanisms represent a complex pathogenesis involving circulatory abnormalities, such as impaired blood volume regulation, cardiovascular deconditioning, sympathetic nervous system hyperreactivity, and autoantibody involvement. Key pathophysiological features of POTS include abnormal peripheral vascular function, partial denervation of the lower extremities, and increased venous pooling, which are associated with decreased circulating blood volume (6).

Patients with POTS experienced a reduction in dizziness while standing, blurred vision, headache, palpitations, and fatigue when wearing an abdominal compression band. Diedrich et al. reported that venous retention in the abdominal vessels during standing is two to three times greater than that in the lower extremities (17). It is speculated that the abdominal compression band reduces blood retention in the abdominal veins and improves hemodynamics by maintaining the circulating blood volume, which contributes to symptom relief while standing. The mechanism of headache in patients with POTS is unclear, although cerebral hypoperfusion may be a potential cause of orthostatic headache (18). We hypothesized that improved hemodynamics contributed to headache relief.

Significant improvements were also observed in hemodynamic parameters, with the abdominal compression band increasing changes in CI during standing, suppressing decreases in SV and increases in HR, and reducing decreases in ID. In the standing position, the abdominal compression band stabilized blood circulation, potentially alleviating the significant HR increase characteristic of POTS.

Conversely, in the supine position, decreased CI and SV, and increased TPR and HR were observed, possibly due to partial inhibition of venous return.

The band pressure was set at 20 mmHg, which is significantly higher than the venous pressure in the abdomen (approximately 5.5 mmHg) (19). The band pressure was also set at 20 mmHg in this study, based on reports that the compression garment should be worn at 20–30 mmHg to increase venous return (20, 21) and a previous study by Tanaka (10), where 20 mmHg was used. This indicates the need to adjust the band pressure to suit the hemodynamics of each patient.

The CI and SV in the supine was lower with the band. There was no significant difference in CI when standing with or without the band, but SV was larger when standing with the band. These results show that venous return while standing was maintained by wearing the band. CI is affected by HR, but the HR was significantly higher without the band, so we consider that there was no significant difference in CI while standing with or without the band.

The frequency analysis of autonomic nervous system function showed that wearing the abdominal compression band tended to decrease cardiac parasympathetic function and increase cardiac sympathetic function in the supine position. In the supine position, wearing the abdominal compression band decreased venous return, which decreased parasympathetic activity via the vagus nerve by decreasing the intraventricular pressure in the right atrium. However, the decrease in cardiac output due to reduced venous return potentially caused a decrease in blood pressure, triggering sympathetic activation in response to high-pressure system receptors.

Notably, the decrease in the cardiac parasympathetic function was alleviated while standing. Although a decrease in cardiac parasympathetic function caused an increase in HR, the increased venous return and hemodynamic stabilization due to the abdominal compression band may have prevented a significant decrease in cardiac parasympathetic function, preventing an uncontrolled increase in HR.

These findings support the efficacy of the abdominal compression band as an adjunctive therapy, highlighting its advantages in improving hemodynamics and enhancing autonomic nervous function in children with POTS. Because some participants reported discomfort with the abdominal compression band, we recommend that it be worn while symptoms occur, rather than throughout the day. The study suggested that effective use of the abdominal compression band is likely to contribute to an improvement in patients' quality of life (QOL).

## 5 Limitation

This study has some limitations. First, further studies are needed to determine whether the band pressure setting is appropriate for achieving an effective result for each patient. Second, this study focused on the short-term effects and not on the long-term effects on patient health. Third, previous studies have reported greater symptomatic improvement with beta-blockers compared with the abdominal compression band alone (22) and additional benefits when combined with physical measures, such as leg crossing (23), which may be a topic for future research. Fourth, assuming that the abdominal compression band increases venous return, cerebral blood flow may be changed. The correlation between the abdominal compression band and cerebral blood flow is another interesting research topic. Fifth, the sample size of this study was small and was not compared to healthy participants. Future studies should increase the sample size and make comparisons with healthy participants. Sixth, there are racial differences in autonomic nervous function (24). This study used standardized diagnostic criteria for pediatric POTS in Japanese patients (The diagnostic criterion for pediatric POTS in Europe and the United States is an increase in heart rate of 40 bpm or more, while in Japan it is 35 bpm or more.). Similar studies in other countries are awaited.

## 6 Conclusion

The abdominal compression band has shown the potential to relieve symptoms while standing and improve hemodynamics and autonomic nervous function in children with POTS.

## Data Availability

The datasets presented in this study can be found in online repositories. The names of the repository/repositories and accession number(s) can be found in the article/Supplementary Material.
